# Ancestry Analysis in the 11-M Madrid Bomb Attack Investigation

**DOI:** 10.1371/journal.pone.0006583

**Published:** 2009-08-11

**Authors:** Christopher Phillips, Lourdes Prieto, Manuel Fondevila, Antonio Salas, Antonio Gómez-Tato, José Álvarez-Dios, Antonio Alonso, Alejandro Blanco-Verea, María Brión, Marta Montesino, Ángel Carracedo, María Victoria Lareu

**Affiliations:** 1 Forensic Genetics Unit, Institute of Legal Medicine, University of Santiago de Compostela, Santiago de Compostela, Galicia, Spain; 2 Genomic Medicine Group, CIBERER, University of Santiago de Compostela, Santiago de Compostela, Galicia, Spain; 3 University Institute of Research Police Sciences (IUICP), DNA Laboratory, Comisaría general de Policía Científica, Madrid, Spain; 4 Faculty of Mathematics, University of Santiago de Compostela, Santiago de Compostela, Galicia, Spain; 5 Instituto Nacional de Toxicología y Ciencias Forenses, Delegación de Madrid, Spain; Institut Pasteur, France

## Abstract

The 11-M Madrid commuter train bombings of 2004 constituted the second biggest terrorist attack to occur in Europe after Lockerbie, while the subsequent investigation became the most complex and wide-ranging forensic case in Spain. Standard short tandem repeat (STR) profiling of 600 exhibits left certain key incriminatory samples unmatched to any of the apprehended suspects. A judicial order to perform analyses of unmatched samples to differentiate European and North African ancestry became a critical part of the investigation and was instigated to help refine the search for further suspects. Although mitochondrial DNA (mtDNA) and Y-chromosome markers routinely demonstrate informative geographic differentiation, the populations compared in this analysis were known to show a proportion of shared mtDNA and Y haplotypes as a result of recent gene-flow across the western Mediterranean, while any two loci can be unrepresentative of the ancestry of an individual as a whole. We based our principal analysis on a validated 34plex autosomal ancestry-informative-marker single nucleotide polymorphism (AIM-SNP) assay to make an assignment of ancestry for DNA from seven unmatched case samples including a handprint from a bag containing undetonated explosives together with personal items recovered from various locations in Madrid associated with the suspects. To assess marker informativeness before genotyping, we predicted the probable classification success for the 34plex assay with standard error estimators for a naïve Bayesian classifier using Moroccan and Spanish training sets (each n = 48). Once misclassification error was found to be sufficiently low, genotyping yielded seven near-complete profiles (33 of 34 AIM-SNPs) that in four cases gave probabilities providing a clear assignment of ancestry. One of the suspects predicted to be North African by AIM-SNP analysis of DNA from a toothbrush was identified late in the investigation as Algerian in origin. The results achieved illustrate the benefit of adding specialized marker sets to provide enhanced scope and power to an already highly effective system of DNA analysis for forensic identification.

## Introduction

On the 11th of March 2004 ten improvised explosive devices (IED) were detonated on four commuter trains entering Madrid during the morning rush hour in a coordinated attack that killed 191 people and injured 1,755. DNA analysis supporting the subsequent investigation centered on standard forensic tests: autosomal and Y-chromosome STR profiling of 226 reference samples and more than 600 exhibits. The exhibits analyzed were predominantly contact traces that included fragments of detonated IEDs, suspect vehicles, an unexploded IED found at El Pozo train station and personal items recovered from bomb assembly sites or houses used by suspects. STR analysis used Amp*fl*STR Identifiler™ in all cases and Y-filer™ in a majority of cases, typing 15 autosomal loci plus amelogenin and 16 Y-chromosome loci respectively. STR typing was also supplemented with standard mtDNA sequence analysis of a majority of exhibits. Seven complete STR profiles, originating from five personal items together with a handprint on the handle of the bag containing the undetonated El Pozo IED, failed to match any of the suspects held at the start of the court case in February 2007. Following an order from the Presiding Judge, the seven unmatched DNA extracts became the focus of specialist genotyping to attempt assignment of ancestry. The judge's direction specified that ancestry analysis should be confined to the comparison of European with North African variability in order to differentiate these two population groups alone.

As quantities of DNA remaining from the initial STR analysis were very limited, an important pre-amble to the extended genotyping was estimation of likely success in assigning ancestry, i.e. choosing the approach that gave the minimum misclassification error from amongst the different ancestry marker sets available. The loci we opted to assess to differentiate Europeans from North Africans were: autosomal AIM-SNPs based on an established 34plex assay [1], mtDNA coding region SNPs and Y-chromosome SNPs. The Identifiler™ STRs were not used by us to differentiate the two population groups as these loci were considered less accurate for assigning ancestry than dedicated AIM-SNPs [2]. Furthermore, although mtDNA and Y-chromosome markers routinely demonstrate informative geographic differentiation, the comparison of uni-parental marker sets, if chosen, would require particular care since the two regions have a proportion of shared mtDNA and Y-chromosome lineages resulting from past gene-flow across the western Mediterranean [3]–[21]. However it should be noted that estimates of the extent to which population movements have contributed to patterns of variation in this region differ between markers and amongst studies [4]–[21]. In addition, mitochondrial DNA and the Y-chromosome represent only two independent loci in the genome, so their power to estimate the ancestry of an individual is lower than a large set of highly differentiated markers such as AIM-SNPs distributed widely amongst the autosomes that can freely recombine and segregate. The 34 AIM-SNPs in the ancestry assay we used are located across twenty autosomes.

A simple Bayesian classifier analyzing the autosomal AIM-SNPs allowed an assessment of their ability to differentiate Europeans and North Africans by cross validation of training set samples used in the likelihood calculations. This permitted a review of classification error: how many training set samples were incorrectly assigned, and classification performance: the range of probabilities observed ahead of deciding the test priorities for scant case material. The classification error estimators of sensitivity and specificity, common to genetic association studies, simply correspond in this case to European and North African classification success in an arbitrary link to each population group.

## Materials and Methods

### Ethics Statement

Population reference samples had been voluntarily provided at a previous date by residents of the Madrid metropolitan area with Spanish or Moroccan ancestry. Buccal swabs were collected by the Comisaria General de Policia Cientifica, Madrid with written, informed consent. Donors were informed the samples would be anonymized and used to derive allele frequency data representative of their population of origin. They verbally declared the population of origin of their parents to be either wholly Spanish or Moroccan and gave signed confirmation they understood the circumstances of sample use. DNA sets were stored with numbers and population labels only, at no point were numbers assigned to names from the consent forms. The University of Santiago de Compostela ethics committee gave institutional approval for the study.

### Samples analyzed

Training sets were made from the population reference samples collected as described above and comprised 48 DNAs each from Spanish and Moroccans resident in Madrid.

Ancestry analyses were made of DNA extracted from the following forensic exhibits:

Sample 1: Contact traces recovered (by swabbing) from a razor blade collected from the ruins of the Leganés flat occupied by a large group of suspects and subsequently destroyed by explosions triggered by the occupants.

Sample 2: Contact traces recovered (by swabbing) from a handprint on the handle of a bag found at El Pozo station containing an undetonated IED.

Sample 3: Buccal cells recovered (by cutting bristles) from a toothbrush collected at Leganés.

Sample 4: Contact traces recovered (by cutting) from a blanket in a house occupied by a suspect.

Sample 5: Contact traces recovered (by swabbing) from a woolen hat collected in the house where the bombs were assembled.

Sample 6: Contact traces recovered (by cutting) from a scarf collected in a van used by suspects to go to one of the train stations attacked. The only female DNA of the seven extracts.

Sample 7: Contact traces recovered (by cutting) from the same scarf as sample 6.

### Assessment of Bayesian classifier error and performance

Estimation of likely ancestry assignment error from each marker set was based on the observed distribution of variability between Europeans and North Africans either directly, by genotyping the 34 AIM-SNPs in samples of 48 individuals each from Spain and Morocco, or indirectly by reference to reported haplotype frequencies from previous population surveys of the Y chromosome [4]–[16] and mtDNA [17]–[21]. The advantages of genotyping dedicated sample sets from each population for the AIM-SNP assay were: i. custom training sets could be made for the classifier and: ii. the divergence between the populations could be measured by cross validation analysis (i.e. re-classifying each training set member using the reconfigured one-out set). Cross validation of the training sets proved valuable ahead of deciding how to genotype limited case material as it allowed an assessment of both the accuracy and performance of the AIM-SNP ancestry test. Accuracy was measured from the misclassification error - the proportion of individuals whose ancestry predicted by the test did not match their self-declared ancestry, while performance was gauged from the range of assignment probabilities observed across each training set. We have found that two populations that are closely related due to admixture or recent shared ancestry can be more easily assessed by ranking the classification likelihoods of training sets in a combined plot of both populations. This method shows any outlying individuals with markedly lower assignment probabilities than the training set as a whole that can arise from above-average levels of mixed ancestry in the individuals. At the same time it is possible to review the range of assignment likelihoods amongst those misclassified in cross validation for comparison with de-novo samples which may also fall within this range due to significant mixed ancestry. Consequently, having opted to use the 34plex AIM-SNP assay as the principal system of analysis to obtain ancestry assignments for all seven case samples we interpreted each classification probability with reference to the training set probability range. While four samples had very high ancestry probabilities, the other three were interpreted to show insufficiently high probabilities to permit a secure assignment. The inference made was that these individuals were more likely to be from North Africa than Europe but with higher levels of mixed ancestry than the majority of training set samples.

### AIM-SNP genotyping and statistical analysis

The single base extension assay conditions, multiplex details and primer sequences were as previously described in detail [1]. The dye-linked primer extension products were detected using an Applied Biosystems 3100 sequencer and SNP alleles were assigned based on a previously validated +/− 0.5 bp mobility window. DNA samples comprised aliquots of the extracts previously used for STR typing and ranged from ≤0.1 ng/ul to 3.3 ng/ul based on standard Quantifiler™ forensic DNA quantitation tests.

From the 34-SNP profiles obtained a naïve Bayes classifier was used to make ancestry assignments as previously detailed [1], where the independence of the 34 SNPs providing the variable parameters had already been established. In this system maximum likelihood estimates of the probability of ancestry from one of a set of populations equate to the joint allele frequency distributions derived from training sets analyzed by the classifier. Since SNPs are predominantly binary polymorphisms relatively small samples of 40–50 individuals from each class (population) provide representative training sets.

The classification algorithm assigns an individual (ind) to the population from pop_i_ …pop_n_ that maximizes the posterior probability:
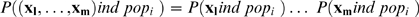
(1)where (x_l_,…,x_m_) is the SNP genotype profile.

**Figure 1:**
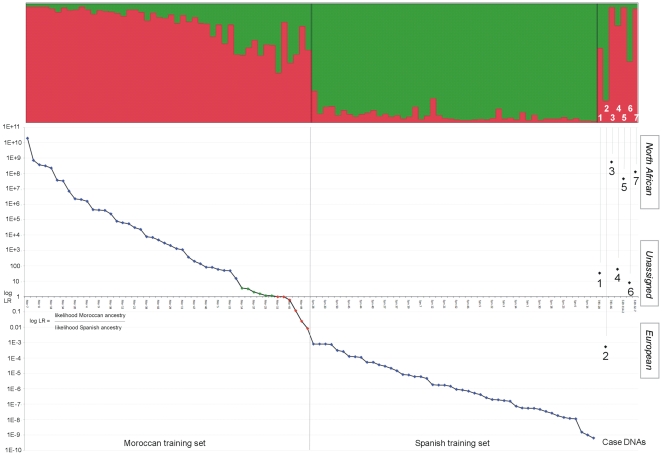
Plot of ranked assignment probabilities obtained from cross validation of training sets. Probabilities of case samples are shown on the right as black diamonds with reported ancestry assignments shown next to the values. (case sample numbers as listed in Materials and Methods). LR is a log scale of the likelihood ratio of Moroccan classification probability/Spanish classification probability, so values below the mid-line of 1 (i.e. balanced odds) are reciprocal values and the distance from the midline denotes higher probability of ancestry from one of the two populations. Red diamonds show four Moroccans misclassified as Spanish and two with balanced odds of 1, green diamonds six Moroccans exhibiting low probabilities compared to the population sample as a whole. The STRUCTURE analysis plot (K = 2) is aligned matching each component sample below.

Unobserved alleles default to frequency 1/(2n+1) where n is training set sample size. The above algorithm is imbedded in an open-access web portal (http://mathgene.usc.es/snipper/) accepting custom training sets and classifying profile submissions based on a user's own choice of SNPs and populations (hyperlink: *‘classification with a custom Excel file of populations’*). Assignment probabilities are given as −log likelihoods to ease handling of large differences in values. Misclassification error estimation options for uploaded training sets (hyperlink: *‘thorough analysis of population data with custom Excel file’*) include re-classification (alternatively termed: apparent error), verbose or non-verbose cross validation and bootstrap error. We used re-classification and verbose cross validation error estimators to assess the classification accuracy of the 34 AIM-SNPs for the two training sets. The complete 34-SNP training set profiles are available in supporting information, Table S1. The assignment probabilities estimated from one-out cross validation of 48 Moroccan and 48 Spanish training set samples were plotted in the combined chart shown in Figure 1. A single value: the log likelihood ratio of probability of Moroccan ancestry over probability of Spanish ancestry was plotted for the vertical axis for both sample sets, so values obtained for Spanish samples are the reciprocal of the Moroccan values and a midline value of 1 represents balanced odds of ancestry between each population. Although this plot detected classification probability outliers amongst the Moroccan samples, all 48 samples were retained in both the training sets used to assign ancestry to the case samples.

In order to compare the above classifier with an established alternative system of ancestry assessment we performed STRUCTURE analysis [22] of the 96 training set samples and the seven case samples. We used the 34 SNP genotype data and two inferred populations (K = 2) to assess the level of mixed ancestry detectable in the training set samples and to evaluate the degree to which outlying individuals shared patterns of allelic variability with the population sample as a whole. STRUCTURE runs consisted of 200,000 Markov Chain steps after a burn-in of length 200,000 with five replicates. Other runs with K values above 2 were made but did not produce coherent patterns of group membership. Although STRUCTURE and our web-based classification algorithm both offer an approach to classifying SNP profiles based on Bayes principles, the web portal was designed specifically for forensic use as it allows the analysis of single profiles in real time while providing the flexibility to change or re-configure training sets and link them back to the analysis of unknown profiles, also in real-time. STRUCTURE incorporates useful features that compliment the web portal (e.g. allowing the classification of individuals in *K* groups without *a priori* knowledge related to the allocation of samples into population groups) but is computationally demanding. In contrast, classification performance for user defined SNP sets and populations can be obtained in seconds using the web portal.

### Y Chromosome SNP genotyping

Five of the six male DNA samples had sufficient DNA left after AIM-SNP analysis to genotype Y-chromosome SNPs. A total of 21 Y-SNPs were genotyped using 3 multiplexes (multiplex 1: SRY10831, M213, M9, M70, M22, Tat, 92R7, M173, P25, multiplex 2: M210, M170, M26, M304, M172, M62, multiplex 3: M96, M35, M78, M81, M123, M34). Multiplex details, primer sequences and PCR conditions are as previously detailed [12].

As the five samples tested by Madrid with Y-STRs could be allocated into Eurasian haplogroups the choice of Y-SNP multiplexes was largely dictated by these prior results. Furthermore the Y-SNP multiplexes allowed a greatly improved resolution of the most common Eurasian haplogroups found on each side of the Mediterranean. Multiplex 1 analyzes SNPs more informative for European haplogroups and Multiplex 3 those more informative for North African haplogroups. Supporting information Table S3 includes the most recent Y-SNP based haplogroup designation tree used to assign the five typed samples to their haplogroups.

### MtDNA genotyping

The two mtDNA hypervariable regions, HVS-I and HVS-II were sequenced for nucleotide ranges: 16024–16365 and 72–340, following previously described protocols [23]. Nomenclature of mtDNA variants is based on the revised Cambridge reference sequence [24].

### Use of expanded training sets to reassess the original AIM-SNP case sample ancestry assignments

Forty-eight Moroccan and Spanish individuals were originally available to us to make training sets. We also considered using a single North African population, termed Mozabite, included in the CEPH human genome diversity panel (CEPH-HGDP). The CEPH Mozabite samples were not used as these were considered to be a population much more divergent from Europe than others in this region. However training sets should ideally sample as much regional variation as possible. Therefore to reassess the original assignments made for the 11-M investigation we collected an additional 67 Spanish together with a broader North African sampling of: 97 Tunisians (three different regions), 35 Algerians and 31 Libyans with 29 CEPH Mozabite also included. The expanded training sets of 115 Spanish and 240 North Africans were then used in a fresh analysis to assign ancestry to the case profiles. The addition of extra reference populations also gave a well differentiated principal component analysis (PCA) plot based on two and three components. The PCA analysis had originally been attempted with the 48-sample training sets but the differentiation of Europeans and North Africans and case samples was not clear in some parts of the plot, notably in the middle of the two adjoining clusters.

## Results

### Accuracy and performance of the AIM-SNP classifier

The results of the statistical assessment of the AIM-SNP classification accuracy from re-classification and cross validation are shown in Table 1. Re-classification analysis: taking each sample in turn and classifying it using the unmodified training set gave 100% success in each population. Cross-validation analysis: classifying each sample with a training set modified to exclude only that sample gave 100% success for Europeans, while four Moroccans were misclassified as European and two had balanced odds of 1: giving 88% success in the assignment of North African ancestry. Because the four Moroccan samples that were misclassified also gave the four lowest classification probabilities to be European and all cross-validated European samples gave high probabilities, the accuracy of the AIM-SNP test was considered high enough to make this the best approach for the limited case material in preference to mtDNA and Y-chromosome SNP analysis.

**Table 1:** Reclassification and cross validation analysis of training sets.

**Reclassification success**	European	North African
Spanish training set classified as:	100%	0%
Moroccan training set classified as:	0%	100%
**Cross Validation**	European	North African
Spanish training set classified as;	100%	0%
Moroccan training set classified as:	12%	88%

Although the autosomal AIM-SNP genotyping assay amplified sub-optimal quantities of DNA, extracted mainly from contact traces on touched objects, 33 of 34 SNPs were successfully typed in all seven case samples. The SNP rs727811 was not detected in all tests performed due to problems with the extension primer that had not been corrected prior to receipt of the case material. Representative single base extension SNP genotyping profiles from four of the case samples are shown in supporting information, Figure S1.

### Variability of the 34 AIM-SNPs in European and North African populations

Due to the problem with rs727811 outlined above the training sets and their analyses excluded this SNP. Training set allele frequencies were estimated and compared with previously published data [1] from eight widely distributed European populations or the Mozabite sample of the CEPH-HGDP. The CEPH European populations originate from: Orkney Islands in Scotland, France, the French Basque region, Bergamo in North Italy, Tuscany, Sardinia, northwestern Russia and Adygea in southeastern Caucasus, while Mozabite population is from the M'zab region of Central Algeria. All CEPH-HGDP allele frequencies for the 34 SNPs are available online at the open-access SPSmart SNP browser (http://spsmart.cesga.es/snpforid.php) [25]. The allele frequencies from these nine populations were used to assess the training sets by calculating the divergence metric: *I_n_* [26] for the Spanish and the CEPH-HGDP European populations compared to Moroccans and Mozabites. Table 2 lists the *I_n_* values obtained and indicates a trend of mildly decreasing divergence between Europe and North Africa along an approximate NW to SE geographic axis in Europe. The divergence between Spanish and Moroccan samples is slightly higher than expected given the degree of shared mtDNA and Y-chromosome variability previously reported for these populations, but is in line with other divergence levels shown here by three mid-European populations from France, the French Basque region and North Italy. The Mozabites are more divergent from Europe than the Moroccans and this probably reflects lower levels of admixture with Europe since this population has a more isolated regional distribution amongst oases of the northern Sahara. Therefore the choice of a Moroccan training set to analyze differentiation between Europe and North Africa may take more account of the effects of admixture across the west Mediterranean than an initial plan to use the Mozabite sample as a training set. Finally, it has recently been inferred from analysis of X-chromosome SNPs that Moroccans are more genetically distant from other more homogenous populations of the North African Mediterranean region [27], with the authors of this study speculating that this was due to more frequent recent migrations in northwest Africa and a higher rate of movement amongst females.

**Table 2:** Cumulative divergence values for pairwise comparisons between nine European populations and two North African populations.

	*I_n_* with Morocco	*I_n_* with Mozabite
Orkney Islands	1.380	1.532
Spain	1.078	1.428
France	1.056	1.421
French Basque	0.987	1.318
Bergamo, Italy	0.911	1.145
Russia	0.800	1.159
Tuscany, Italy	0.759	0.925
Adygei	0.614	0.773
Sardinia	0.538	0.863
*I_n_* Spain:6 CEPH-HGDP European populations*		0.056
*I_n_* Morocco:Mozabite		0.317

Values below show within-population group divergence estimates.

* Sardinian and Adygei CEPH-HGDP populations excluded.

In order to further assess the representativeness of the training sets we calculated divergence between the Spanish and a combined group of six CEPH-HGDP European populations (based on previous analyses [1] the outlier Sardinian and Adygei populations were excluded), together with divergence between Moroccan and Mozabite populations. These within-group *I_n_* values are included at the bottom of Table 2 and show that variation between Spanish and other European populations represents just 6% of the average divergence between Europe and Morocco, but between Moroccan and Mozabite populations reaches 35% of the average divergence between Europe and Morocco, although, as described above, Mozabites are likely to be an unrepresentative population sample of the Algerian region.

AIM-SNP allele frequency estimates from the training sets and divergence values from pairwise comparisons with equivalent populations are listed in supporting information Table S2. As points of reference allele frequencies are also given for Mozabite, the six combined CEPH European populations and HapMap Utah residents with north and west European ancestry (CEU), although HapMap has not characterized seven of the 34 SNPs. The most divergent SNP between Spanish and the six CEPH Europeans is rs182549; a marker associated with hypolactasia and the LCT gene [28] that shows the highest levels of variability within Europe of any genomic region studied to date [29].

In summary, the Spanish training set provides a very suitable proxy for European variation as a whole, while the more restricted comparison of the Moroccan training set with the Algerian Mozabite indicates much higher divergence although this is likely to partly reflect differences in levels of admixture with Europe and the character of the two North African regions. Overall selecting an adequately representative North African training set is more difficult than the equivalent process for Europe, but we took a conservative approach by choosing the population showing a lower divergence with Europe. These limitations were in part overcome by a later re-analysis of case profiles using much expanded training sets with the principal benefit of taking a more full account of allele frequency variation across North African regions.

### AIM-SNP ancestry assignments

The ranked AIM-SNP training set assignment probabilities, case sample probabilities and matching STRUCTURE patterns for K = 2 [22] are shown in a combined plot in Figure 1. Both training sets show similar probability ranges for the majority of individuals: from 100–1,000 times more likely up to 10 million times more likely to be European or North African. The probabilities in Figure 1 are based on a likelihood ratio between the two population groups sampled to compile the training sets, so the possibility of an individual having ancestry outside of Europe or North Africa, for example from sub-Saharan Africa, are not precluded by any of the values obtained. However, the investigation specifically directed that a pairwise comparison be made between two population groups, therefore only assignments to one of these two groups were calculated and reported. Despite showing similar classification probability ranges a clear difference is evident between the distributions of Moroccan probabilities and those of the Spanish. No Spanish probability falls below 1,000 times more likely to be European, while six Moroccan samples were misclassified as European (red points below or on the midline) and a further six samples had near balanced odds to be European or North African (green points just above the midline). Amongst these twelve classification outliers eleven showed the highest levels of joint group membership in the STRUCTURE analysis suggesting that probabilities less than 100 times more likely to be European or North African required interpretation with caution. This is underlined by the close match between STRUCTURE patterns and assignment probabilities observed in the case samples with two of the three SNP profiles falling into the outlying probability area (below 100 times more likely to be North African) showing equivalent levels of joint group membership.

### Collating results into an ancestry assignment report

The AIM-SNP 34plex assay was run in duplicate with fully concordant results and enough DNA extract remained to perform Y-chromosome SNP marker analysis for five of the six male samples. Table 3 shows the statistical assessments and ancestry predictions for each case sample reported to the investigation based primarily on AIM-SNP likelihoods although an assessment was also made of the uni-parental marker analysis, using published data of Y-chromosome [4]–[16] and mtDNA [17]–[21] variability in each region. The full genotyping results from Y-chromosome and mtDNA markers and their interpretation are outlined in supporting information Table S3. Only sample 3 gave different indications of ancestry between AIM-SNPs and uni-parental markers and is discussed in the next section. A distinction emerges amongst case samples from their AIM-SNP probabilities shown in Figure 1 and Table 3: three profiles give very strong indications of North African ancestry in the range 45–560 million times more likely, three profiles that were not assigned give weak indications of North African ancestry but within the outlying probability area i.e. below 100 times more likely, and a single profile gives a probability of 1,700 times more likely to be European. The last of these was interpreted to be a reliable European assignment despite being at the lower end of the likelihood range shown by the Spanish training set.

**Table 3:** Ancestry assignment probabilities from AIM-SNP analysis using a Bayes classifier (output: -log likelihoods), derived likelihood ratios (LR) and ancestries reported to the investigation.

case sample	-log likelihood: North African	-log likelihood: European	LR North African	LR European	Ancestry assignment AIM-SNPs	Assessment of ancestry Y/mt loci	Ancestry assignment reported
Razor	9.6948E-15	2.6870E-16	36.08*		-	-	-
Handprint on bag	2.4805E-18	4.259E-15		1,717	European	European	European
Toothbrush	1.6811E-14	2.9613E-23	567,680,319		N African	European	N African
Blanket	9.0947E-17	1.509E-18	60.27*		-	N African	-
Hat	2.4254E-15	5.2772E-23	45,959,585		N African	N African	N African
Scarf profile 1	2.2546E-20	2.4632E-21	9.15*		-	NT	-
Scarf profile 2	1.3096E-19	1.0111E-27	129,513,282		N African	NT	N African

* No assignments were made for the three samples with AIM-SNP assignment probabilities lower than 100× more likely to be North African

NT: not tested.

### Matching of the toothbrush DNA sample to a suspect of Algerian ancestry

Late in the investigation the Presiding Judge ordered samples to be collected from the relatives of missing suspects for whom international arrest warrants had been issued, to help identify unmatched STR profiles found in locations closely associated with the suspects. This allowed the identification of sample 3: DNA recovered from the toothbrush found at a flat in Leganés as belonging to an Algerian man, by using familial searching of a Spanish DNA database. Therefore a single opportunity occurred to compare the known, Algerian, ancestry of this individual with the AIM-SNP classification probability of sample 3: a predicted ancestry of 567 million times more likely to be North African than European. It is notable that this was the only sample that gave slightly contradictory evidence of ancestry from the AIM-SNP analysis and the uni-parental marker analysis. The Y-STR profile obtained belongs to the most frequent Y-chromosome haplogroup in Western Europe: R1b. However, this haplogroup is also frequent in North Africa, accounting for ∼10% of the Algerian population [16]. The mtDNA HVS-I/II profile obtained: 73G 146C 153G 256T 263G 309.1C 315.1C 16182C 16183C 16189C 16193.1C 16223T 16278T can be unequivocally classified as a member of haplogroup X1. Searching the HVS-I haplotype in several population datasets (excluding the highly unstable polymorphisms 16182C 16183C 16193.1C) yields 133 perfect matches, 12% of these matches are found in North Africa and the Middle East while the rest are observed mainly in Europe. Considering the fact that the European database is about ten times larger than those of North Africa and the Middle East combined, it is not possible to reasonably assign this HVS-I haplotype to a particular geographical region. In summary, the mtDNA and Y-chromosome polymorphisms of the toothbrush DNA are found in both population groups but at a lower frequency in North Africans, but the principal difficulty in interpreting these patterns arises from disparities in the size of the databases available. This underlines one of the advantages autosomal AIM-SNPs offer in allowing reliable population variability surveys to be made from relatively small sample sizes compared to uni-parental markers.

### New case profile ancestry probabilities using expanded or modified training sets

As a means to validate the original assignments reported to the investigation we made new ancestry analyses using a Spanish training set expanded two and a half-fold alongside a combined Moroccan, Tunisian, Libyan and Algerian set expanded five-fold. Reclassification and cross validation error were re-estimated and gave similar classification performance compared to the 48 sample training sets used for the casework analyses, error estimates are given in supporting information Table S4. The original and new case sample ancestry probabilities are compared in supporting information Table S5. In all cases the assignments were consistent and the new probabilities obtained were of the same order of magnitude, notably the three samples that were previously not reported to the investigation as North African continued to give inconclusive probabilities.

Additional case profile classifications were made using the original Moroccan training set with outliers removed: excluding the four misclassified individuals (the four lowest red points in Fig. 1) or these four plus the eight samples showing below average likelihoods (two red and six green points close to the midline). As with the expanded training set analyses all case profiles continued to give consistent levels of classification likelihood although sample 6 changed assignment to European using the reduced 36 sample Moroccan training set.

**Figure 2:**
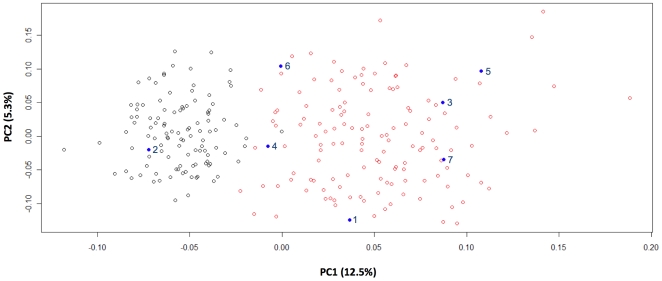
Principal component analysis of case samples and the extended Spanish (black points, n = 127) and North African (red points, n = 240) training sets. PC1 and PC2 % contributions shown in brackets.

Finally, the clearest principal component analysis was obtained using the expanded training sets with improved patterns compared to original attempts using 48-sample sets. The plot of the two principal components is shown in Figure 2 and these accounted for 12.5%, and 5.3% of the total variation.

## Discussion

This report outlines our first experience of applying an autosomal SNP based ancestry test and accompanying statistical tools to a criminal investigation. Two caveats to this approach that were discussed in the original publication [1] are even more important to emphasize in this case. Firstly, the test was designed to analyze the major population groups of Africa, Europe and East Asia. Therefore a thorough assessment of the effects of ascertainment bias on the informativeness of these SNPs for a specialized population comparison was an essential preamble to their use. Secondly, populations on continental margins such as those of the Mediterranean fringe can be expected to show high levels of admixture that will erode divergence and consequently raise the rate of misclassification error.

A large number of autosomal ancestry informative SNP sets have been published and are available to use, primarily to help assess the ancestry or degree of admixture of case-control association study subjects. When originally designing the 34plex assay for forensic use we first selected SNPs from a panel of 3,011 evenly spaced loci chosen to differentiate Africans and Europeans [30]. We next selected SNPs directly from online databases that differentiated East Asians from the other two groups and were optimally positioned in the genome [1]. Therefore the 34plex assay comprised SNPs selected with ascertainment bias for maximizing the differentiation of these three major population groups and would not necessarily include the best SNPs for distinguishing Europeans from North Africans. With this in mind our primary concern before deciding to use the 34plex as the first choice system over uni-parental loci was the estimation of classification success. Success in distinguishing CEPH European and North African samples was low in combination with Africans and East Asians, but reached acceptable levels of ∼90% success differentiating Mozabite Algerians and 100% success with Europeans in exploratory pairwise comparisons made to assess the suitability of the test. In addition to better classification success rates with pairwise population comparisons, the Y-STR genotypes from the case material ruled out African and East Asian ancestry in five of the seven profiles and the Presiding Judge specifically directed the differentiation of just two population groups. Therefore we decided to apply the 34plex test. An important additional feature of the 34plex assay was a known track record of successful typing of typical forensic casework material [31], since many of the first choice AIM-SNPs had been replaced due to poor multiplex performance. This SNP panel is, to our knowledge, the only one available that has been demonstrated to be effective when dealing with highly degraded samples. Development of a dedicated SNP set that could focus on the required differentiation was not practical in the timeframe available to us and would have risked failure with such challenging DNA. We have since addressed ascertainment bias in the 34plex assay by developing complementary assays that are forensically robust and focused on more specific differentiations. These include assays to differentiate Native Americans from the other four continental groups, Oceanians from other groups and a 28plex informative for population groups in the Eurasian trans-continental geographic region: Europeans, North Africans and South Asians. Selection of SNPs for these specific comparisons relies on a less biased approach analyzing full genome-wide coverage of markers (650,000 loci) genotyped in 52 populations of the CEPH-HGDP [32]. The improvement in classification performance that can result from combining two or three small-scale autosomal AIM-SNP panels is outlined in supporting information Table S6. It is interesting to note that the most informative SNP in the 34plex for comparing Europe and North Africa: rs16891982 remains the best marker for the purpose. Reliance on large-scale whole genome SNP arrays to collect ancestry markers also carries some risk of ascertainment bias, for example the two tri-allelic SNPs in the 34plex: rs4540055 and rs5030240 were also amongst the most informative SNPs in our analysis but are not properly characterized by systems designed to detect binary variation at each loci.

Although increased misclassification error from admixture is a real possibility we found that confining the analysis to the pairwise comparison requested by the investigation allowed an informed assessment of the genetic differentiation between each group to be made. The ranking of training set probabilities allowed the range of divergence between populations to be scrutinized and it was then possible to identify a region of the probability plot where assignments carried a higher risk of misclassification error. Such an approach was a key factor in deciding to use AIM-SNPs for the requested population comparison and in making the final ancestry assignments with confidence by not reporting three SNP profiles with the weakest probabilities (case samples 1, 4 and 6). Prior to the analyses described here we had applied the same approach to analyze subjects in a drug response trial with self-declared ancestry to be ‘European-American’ or ‘African-American’. By examining the range of probabilities in both groups we were able to identify subjects showing lower than average ancestry probabilities who could therefore represent individuals with highly admixed parentage and genomic backgrounds (unpublished data). This highlights a widely recognized characteristic of self-declared ancestry: that it is not always an adequate indication of the genetic ancestry detected by a set of markers and their subsequent analysis [33]. Regular use is made of the STRUCTURE grouping algorithm and panels of AIM-SNPs in case-control association studies and drug response trials to identify and exclude subjects with atypical ancestries. Such individuals are comparable to the 12–15 Moroccan training set outliers we observed and shown in Figure 1 to give noticeably higher joint group membership with STRUCTURE analysis. Since our application of the AIM-SNP test to admixed populations required a conservative approach and we could not be prescriptive about the likely ancestry of the population samples used, we decided it was important to keep the training sets intact. Nevertheless, classifying the case samples with modified Moroccan training sets that excluded the outliers had little effect on the classification probabilities obtained, notably those of the three profiles we chose not to report ancestries for.

Given the relatively straightforward framework devised for assessing population variability ahead of genotyping limited DNA, the near-complete SNP profiling of challenging case material (in terms of quantity rather than quality) and the high classification probabilities obtained in four of the seven profiles, we considered the first application of autosomal AIM-SNPs to assist a major investigation to be successful. Understandably there has been continued discussion relating specifically to the origin of the handprint on the bag containing the IED, not least because possible involvement of the Basque separatist group ETA was suspected for a large part of the investigation period and a Spanish mining contractor was ultimately convicted of supplying the explosives used in the attacks. All European suspects in the case were excluded as donors of the STR profile of sample 3. While the source of the DNA remains an open question, one positive outcome of the ancestry analysis we provided has been an overhaul of procedures for handling items recovered from terrorist attacks or major crime scenes in Spain. Amongst the Presiding Judge's concluding remarks at the trial was the comment that any number of people could have handled the bag between collection from the scene and forensic analysis, since it was first thought the bag belonged to a victim.

We were initially cautious in recommending the use of 34 SNPs to differentiate such closely related population groups, particularly as the groups, while occupying different regions, can be realistically considered to be members of the same Eurasian metapopulation [32], [34]. However the results proved to be more informative than we expected, due in large part to the focused population comparisons requested. It is certain that we would now rely on two alternative strategies not available at the time of the analyses and both able to give much better differentiation of the two populations studied. Firstly multiplex sizes can be extended up to 48 SNPs using oligo-ligation chemistry [35] and we are developing this technology to improve the accuracy of autosomal SNP based ancestry tests. Secondly it is possible to apply dedicated AIM-SNP multiplexes designed for specific population comparisons as described above. Use of larger multiplexes or tailored AIM-SNP sets will reduce the error associated with interpretation of ancestry markers, particularly those of the two uni-parental loci, when divergence is eroded by extensive population movements between regions due to their demographic history and geographic proximity.

An important observation in the combined analysis of training sets and case profiles was the generally close match of each sample's classification likelihood and their STRUCTURE cluster plot patterns indicating that each approach can aid interpretation by revealing different perspectives of the same Bayesian assessment of individual ancestry. Our classification approach provides a simple system to obtain profile-by-profile likelihoods that are more difficult to execute with STRUCTURE, while STRUCTURE can reveal patterns of group membership from the cluster plots that indicate the degree of admixture between two sets of reference populations. Therefore we would advocate use of both methods in parallel to fully assess the SNP variability of training sets and de novo samples. The use of principal component analysis provides a third way to compare the position of unknown samples with reference data. Analyzing extended training sets with PCA gave consistent patterns to those of STRUCTURE and our single profile classifier. Similarly we observed a high level of consistency between the ancestry assignments made with the original training sets and those based on expanded Spanish and more broadly based North African sampling. All the newly derived ancestry probabilities were consistent with the original values giving identical orders of magnitude and supporting the original assessments made with more limited training set sampling. The improvement in the North African cross validation probabilities underlines the importance of the widest possible sampling of regional diversity in order to properly prepare a Bayesian classifier. This is particularly important when differentiating closely related population groups that may also show high within-group variation.

Finally, as the forensic genetics community becomes increasingly interested in the development of DNA tests that can assist an investigation with intelligence data supplementary to mainstream STR-based identification, it is worth emphasizing that ancestry analysis when carefully applied alongside STR profiling has the potential to yield information critical for the progress of the investigating team and their search for suspects. Though dealing with admixed populations can mean that an ancestry assignment does not necessarily allow the definition of a series of physical characteristic phenotypes normally more closely associated with ancestry such as skin, hair and eye color. Of particular interest here is the fact that a component marker of the 34plex AIM-SNP assay: rs12913832, was recently shown to be associated with blue eyes in homozygous G form, with the A allele associated with non-blue eyes [36], [37]. Therefore it would now be possible to provide the additional information that the rs12913832 GG genotype of the profile from the scarf (sample 6) could have come from an individual with blue eyes with a ∼90% predictability – providing more useful guidance to the investigation than the ancestry analysis could alone.

## Supporting Information

Figure S1 Example electropherograms of four case samples from single base extension genotyping. Solid colors depict peaks identified as extension products by reference to pre-validated mobility windows with an average range of +/−0.5 bp. Peaks with relatively high signal strength not shown as solid colors (notably in the green channel) represent either random signals or those outside the mobility window of each allele.
(17.69 MB TIF)

Table S1 Excel file of 48 Spanish and 48 Moroccan 34plex training set profiles (excluding rs727811) in valid format for data input to the SNP ancestry analysis portal used (http://mathgene.usc.es/snipper/analysispopfile2.html). Samples are ordered to match the LR values plotted in Figure 1. Worksheet 2 lists expanded training sets that include additional Spanish, Tunisian, Libyan and Algerian samples.
(0.28 MB XLS)

Table S2 Reference allele frequencies and divergence values of 34 AIM-SNPs from Spanish and Moroccan training sets, HapMap CEU, six CEPH-HGDP European populations combined and Mozabite. SNPs listed in order of descending power of differentiation for the population comparison analyzed (i.e. training set divergence).
(0.11 MB DOC)

Table S3 Case sample genotypes and analyses. AIM-SNP profiles are provided in the required format for analysis with the training sets of Table S1 using the same ancestry portal (http://mathgene.usc.es/snipper/analysispopfile2.html).
(0.05 MB XLS)

Table S4 Classification error estimation of extended training sets. Table S4A. Reclassification analysis Table S4B. Cross validation analysis
(0.03 MB DOC)

Table S5 New ancestry assignment probabilities for case profiles using modified 48-sample training sets (excluding 4 or 12 outlier Moroccan samples) and analyses using expanded Spanish and North African training sets.
(0.04 MB DOC)

Table S6 Classification error estimations for combinations of four autosomal AIM-SNP sets using CEPH-HGDP samples as training sets.
(0.03 MB XLS)
