# Supplementary figures and images for: Ancestry Analysis in the 11-M Madrid Bomb Attack Investigation
Source: PLoS One. 2009 Aug 11;4(8):e6583. doi: 10.1371/journal.pone.0006583 (PMC2719087; doi:10.1371/journal.pone.0006583)

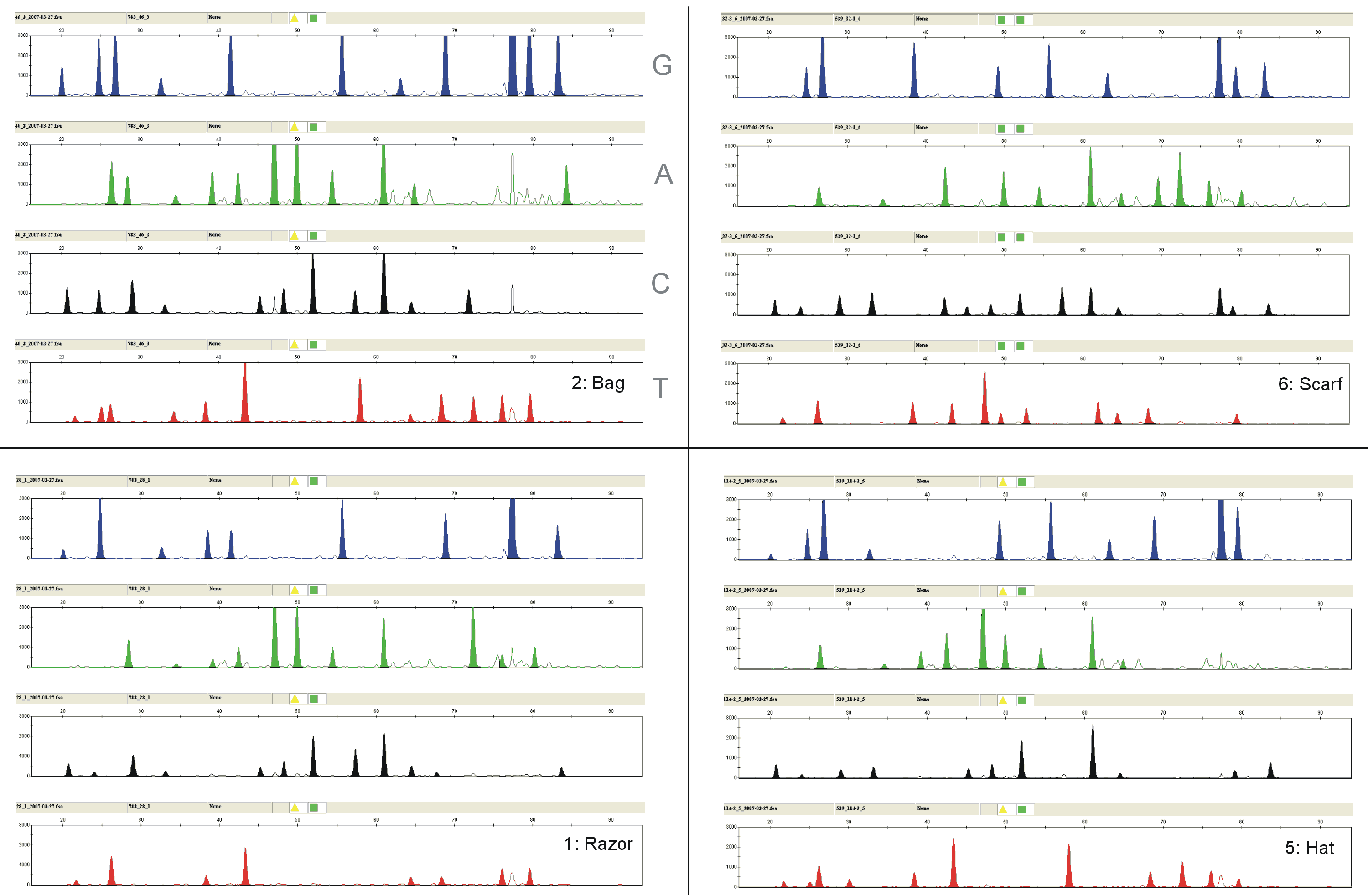

Supplement: Figure S1 — Example electropherograms of four case samples from single base extension genotyping. Solid colors depict peaks identified as extension products by reference to pre-validated mobility windows with an average range of +/−0.5 bp. Peaks with relatively high signal strength not shown as solid colors (notably in the green channel) represent either random signals or those outside the mobility window of each allele. (17.69 MB TIF) [file pone.0006583.s001.tif]
