# Supplementary material for: Ancestry Analysis in the 11-M Madrid Bomb Attack Investigation
Source: PLoS One. 2009 Aug 11;4(8):e6583. doi: 10.1371/journal.pone.0006583 (PMC2719087; doi:10.1371/journal.pone.0006583)
Supplement: Table S2 — Reference allele frequencies and divergence values of 34 AIM-SNPs from Spanish and Moroccan training sets, HapMap CEU, six CEPH-HGDP European populations combined and Mozabite. SNPs listed in order of descending power of differentiation for the population comparison analyzed (i.e. training set divergence). (0.11 MB DOC) [file pone.0006583.s003.doc]

Supporting information, Table S2. Reference allele frequencies and divergence values of 34 AIM-SNPs from Spanish and Moroccan training sets, HapMap CEU, six CEPH-HGDP European populations combined and Mozabite. SNPs listed in order of descending power of differentiation for the population comparison analyzed (i.e. training set divergence).

| AIM-SNP | Ref allele | *I_n_* Spain TS : Morocco TS | HapMap CEU | 6 CEPH EUR populations | Spanish training set | *I_n_* Spain : 6 CEPH EUR | Moroccan training set | Mozabite | *I_n_* Morocco : Mozabite |
| --- | --- | --- | --- | --- | --- | --- | --- | --- | --- |
| rs16891982 | C | *0.1834* | 0.017 | 0.058 | 0.052 | *0.0001* | 0.415 | 0.37 | *0.0010* |
| rs4540055* | T/A | *0.1495* | - | 0.761/0.190 | 0.823/0.156 | *0.0043* | 0.413/0.345 | 0.312/0.448 | *0.0069* |
| rs722098 | A | *0.0799* | - | 0.805 | 0.865 | *0.0032* | 0.5 | 0.672 | *0.0153* |
| rs1335873 | A | *0.0758* | 0.333 | 0.226 | 0.177 | *0.0019* | 0.455 | 0.569 | *0.0066* |
| rs12913832 | A | *0.0589* | 0.208 | 0.411 | 0.489 | *0.0031* | 0.812 | 0.897 | *0.0072* |
| rs5030240* | G/C | *0.0544* | - | 0.705/0.183 | 0.781/0.146 | *0.0041* | 0.232/0.714 | 0.467/0.315 | *0.0860* |
| rs182549 | C | *0.0514* | - | 0.562 | 0.438 | *0.0077* | 0.75 | 0.776 | *0.0005* |
| rs239031 | C | *0.0485* | 0.009 | 0.009 | 0.010 | *0.0000* | 0.177 | 0.172 | *0.0000* |
| rs2572307 | T | *0.0447* | 0.009 | 0.013 | 0.010 | *0.0001* | 0.167 | 0.207 | *0.0013* |
| rs917118 | C | *0.0395* | 0.767 | 0.774 | 0.844 | *0.0039* | 0.594 | 0.534 | *0.0018* |
| rs1886510 | C | *0.0379* | 0.425 | 0.416 | 0.375 | *0.0009* | 0.649 | 0.621 | *0.0004* |
| rs2304925 | T | *0.0377* | 0.750 | 0.773 | 0.854 | *0.0055* | 0.615 | 0.5 | *0.0066* |
| rs773658 | C | *0.0375* | 0.000 | 0.000 | 0.000 | *0.0000* | 0.146 | 0.103 | *0.0021* |
| rs1978806 | A | *0.0306* | 1.000 | 0.995 | 1.000 | *0.0000* | 0.889 | 0.966 | *0.0113* |
| rs2814778 | A | *0.0292* | - | 1.000 | 1.000 | *0.0000* | 0.894 | 0.793 | *0.0097* |
| rs10141763 | T | *0.0274* | 0.948 | 0.925 | 0.927 | *0.0000* | 0.76 | 0.75 | *0.0001* |
| rs2303798 | G | *0.0234* | 1.000 | 0.996 | 1.000 | *0.0006* | 0.896 | 0.741 | *0.0206* |
| *rs2065982* | T | *0.0201* | 0.958 | 0.956 | 1.000 | *0.0058* | 0.906 | 0.931 | *0.0010* |
| rs1024116 | G | *0.0131* | 0.466 | 0.432 | 0.372 | *0.0019* | 0.533 | 0.483 | *0.0012* |
| rs1573020 | G | *0.0103* | 0.000 | 0.022 | 0.021 | *0.0000* | 0.083 | 0.121 | *0.0019* |
| rs2026721 | A | *0.0078* | - | 0.095 | 0.125 | *0.0012* | 0.219 | 0.172 | *0.0017* |
| rs2065160 | T | *0.0061* | 0.879 | 0.792 | 0.844 | *0.0022* | 0.756 | 0.517 | *0.0310* |
| rs10843344 | C | *0.003* | 0.733 | 0.690 | 0.646 | *0.0011* | 0.719 | 0.828 | *0.0085* |
| rs881929 | G | *0.0027* | 0.625 | 0.486 | 0.490 | *0.0003* | 0.417 | 0.586 | *0.0144* |
| rs1426654 | A | *0.0025* | 1.000 | 0.982 | 1.000 | *0.0018* | 0.979 | 0.862 | *0.0259* |
| rs730570 | G | *0.0023* | 0.158 | 0.152 | 0.160 | *0.0001* | 0.213 | 0.31 | *0.0061* |
| rs1498444 | A | *0.0005* | 0.585 | 0.571 | 0.563 | *0.0001* | 0.594 | 0.586 | *0.0000* |
| rs1321333 | A | *0.0003* | 0.508 | 0.500 | 0.490 | *0.0001* | 0.512 | 0.379 | *0.0089* |
| rs5997008 | C | *0.0001* | 0.992 | 0.903 | 0.854 | *0.0028* | 0.844 | 0.914 | *0.0058* |
| rs7897550 | G | *<0.0001* | 0.700 | 0.770 | 0.823 | *0.0021* | 0.812 | 0.879 | *0.0043* |
| rs3785181 | G | *<0.0001* | 0.925 | 0.951 | 0.958 | *0.0001* | 0.953 | 0.966 | *0.0005* |
| rs896788 | G | *<0.0001* | - | 0.848 | 0.844 | *0.0000* | 0.844 | 0.724 | *0.0106* |
| rs2040411 | G | *<0.0001* | 0.292 | 0.350 | 0.313 | *0.0008* | 0.313 | 0.5 | *0.0183* |
| rs727811 | C |  | 0.400 | 0.442 |  |  |  | 0.554 |  |

*Tri-allelic SNPs. Frequencies are given for the two alleles listed by HapMap.

Ref allele: reference allele listed by HapMap or dbSNP. Missing values in HapMap CEU denote seven SNPs not characterized by HapMap to date.

*I_n_*: divergence values (in italics) from comparisons of Spanish:Moroccan training sets (TS), Spanish:six CEPH European populations combined (Sardinian and Adygei excluded) and Moroccan:Mozabite.
