# Supplementary material for: Ancestry Analysis in the 11-M Madrid Bomb Attack Investigation
Source: PLoS One. 2009 Aug 11;4(8):e6583. doi: 10.1371/journal.pone.0006583 (PMC2719087; doi:10.1371/journal.pone.0006583)
Supplement: Table S4 — Classification error estimation of extended training sets. Table S4A. Reclassification analysis Table S4B. Cross validation analysis (0.03 MB DOC) [file pone.0006583.s005.doc]

Supporting information, Table S4. Classification error estimation of extended training sets

Table S4A. Reclassification analysis

| Classified as: | European | North African |
| --- | --- | --- |
| Spanish training set | 100% | 0% |
| North African training set | 3% | 97% |

Table S4B. Cross validation analysis

| Classified as: | European | North African |
| --- | --- | --- |
| Spanish training set | 85% | 15% |
| North African training set | 1% | 99% |
