# Supplementary material for: Ancestry Analysis in the 11-M Madrid Bomb Attack Investigation
Source: PLoS One. 2009 Aug 11;4(8):e6583. doi: 10.1371/journal.pone.0006583 (PMC2719087; doi:10.1371/journal.pone.0006583)
Supplement: Table S5 — New ancestry assignment probabilities for case profiles using modified 48-sample training sets (excluding 4 or 12 outlier Moroccan samples) and analyses using expanded Spanish and North African training sets. (0.04 MB DOC) [file pone.0006583.s006.doc]

Supporting information, Table S5. New ancestry assignment probabilities for case profiles using modified 48-sample training sets (excluding 4 or 12 outlier Moroccan samples) and analyses using expanded Spanish and North African training sets.

|  | **Original 48-training sets** | | **North African 44-training set (less 4 misclassified)** | | **North African 36-training set (less 4 and 8 with low probabilities)** | | | **Expanded training sets** | | |  |
| --- | --- | --- | --- | --- | --- | --- | --- | --- | --- | --- | --- |
| case sample | LR North African | LR European | LR North African | LR European | | LR North African | LR European | | LR North African | LR European | |
| Razor | 36.08 |  | 18.91 |  | | 10.89 |  | | 749.05 |  | |
| Handprint |  | 1,717 |  | 3,074 | |  | 7,512 | |  | 12,204 | |
| Toothbrush | 567million |  | 346million |  | | 847million |  | | 338million |  | |
| Blanket | 60.27 |  | 70.02 |  | | 43.18 |  | | 8.10 |  | |
| Hat | 46million |  | 53million |  | | 87million |  | | 289million |  | |
| Scarf 1 | 9.15 |  | 3.58 |  | |  | 1.82 | | 2.24 |  | |
| Scarf 2 | 129million |  | 173million |  | | 173million |  | | 348million |  | |
